# Epigastric Pain and Fever in a Child

**DOI:** 10.4103/1319-3767.80390

**Published:** 2011

**Authors:** Intezar Ahmed, Sunita Singh, Naveen Chandra, Shiv N. Kureel

**Affiliations:** Department of Pediatric Surgery, CSM Medical University, Lucknow, India

An eight-year-old boy was admitted for evaluation of upper abdominal pain and fever. One year before the admission, he had an episode of subacute intestinal obstruction, which was managed conservatively. During current admission; on physical examination, we noticed a vague upper abdominal nontender distention but no definite palpable abdominal lump. Hemogram was within normal limit except leucocytosis. Serum amylase was markedly raised, and serum lipase was marginally increased. X-ray abdomen erect was suggestive of ground glass appearance with scanty amount of air in abdomen and without any pathological air-fluid level. Ultrasound abdomen showed fluid collection in between the stomach and pancreas. Computer tomography with the administration of oral contrast medium revealed a cavity in hepatogastric fossa with an air-fluid level. The gastrointestinal contrast material had not entered the cavity [Figure 1].

**Figure 1:**
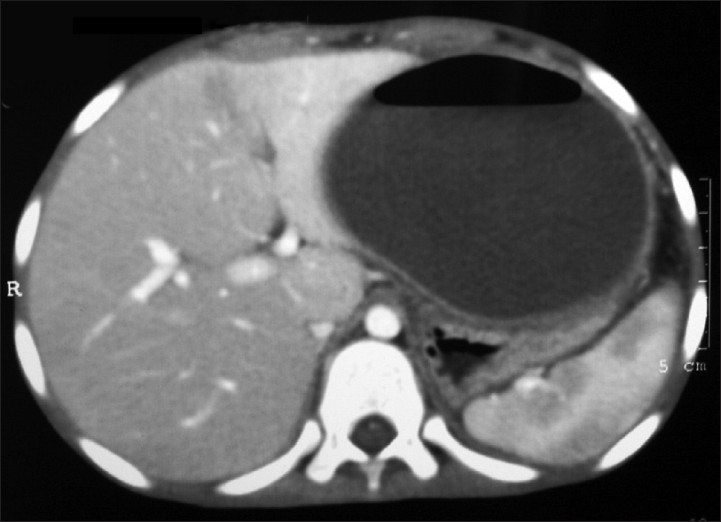
Axial view of computed tomography scan abdomen showing an air-fluid level containing cavity in between liver and stomach

## QUESTION

What is the diagnosis?

## ANSWER

The computer tomography findings were consistent with a subhepatic abscess cavity with an air-fluid level. There was no visible communication with the bowel. Patient had managed by percutaneous drainage of pus cavity under intravenous antibiotic cover and he responded well to the treatment.

Despite advances in surgical technique and medical treatment, intra-abdominal abscesses remain a common diagnostic problem. An air-fluid level may indicate the presence of a fistulous communication to the gastrointestinal tract, but its absence does not necessarily mean there is no communication.[1]

Gas may appear as bubbles in deep, if the medium is thick. In cases of thin material, gas may rise to the surface, forming either superficial bubbles or air-fluid level. Distribution of gas in an intra-abdominal abscess is associated with drainability. Abscesses with superficial gas (superficial bubbles or air-fluid levels) have a greater chance of being drained successfully than do abscesses with deep trapped gas.[2]

In abscesses with classic clinical features and focal abdominal tenderness, ultrasound is a rapid and sensitive diagnostic technique. Enteric communication with abscesses has been shown by barium studies and more recently by CT.[3] The degree of drainability, however, cannot be assessed with these techniques, the use of indium labeled leukocytes scanning is a novel yet logical approach to overcome this problem.

Mortality may reach 100% without proper diagnosis and abscess drainage, despite antibiotic therapy.[3]
